# GENOME-WIDE ASSOCIATION META-ANALYSIS OF PHYSICAL PERFORMANCE IN OLDER ADULTS

**DOI:** 10.1093/geroni/igac059.855

**Published:** 2022-12-20

**Authors:** Adam Santanasto, Douglas Kiel, David Karasik, Ryan Cvejkus, Mary Biggs, Kathryn Lunetta, Joanne Murabito, Joseph Zmuda

**Affiliations:** School of Public Health, University of Pittsburgh, Oakmont, Pennsylvania, United States; Harvard Medical School, Boston, Massachusetts, United States; Hebrew SeniorLife, Roslindale, Massachusetts, United States; University of Pittsburgh, Pittsburgh, Pennsylvania, United States; University of Washington, Seattle, Washington, United States; Boston University School of Public Health, Boston, Massachusetts, United States; Boston University, Boston, Massachusetts, United States; University of Pittsburgh, Pittsburgh, Pennsylvania, United States

## Abstract

Although measures of physical function such as walking speed and time to complete chair-rises are highly heritable, the genetic architecture underlying these phenotypes remains poorly defined. To identify potentially novel genes and pathways underlying physical performance in older adults, we conducted a genome-wide association meta-analysis of the short physical performance battery (SPPB) (Score 0-12) and one of its components, chair-rise time (seconds) in 24,033 Caucasian adults aged 60+ from 13 cohorts (mean cohort age 66.2 ± 5.3 to 84.3 ± 4.1 years; 56.5% women). Cohorts had a genome wide scan imputed to either the Haplotype Reference Consortium or Trans-Omics for Precision Medicine imputation panels. Single nucleotide polymorphism (SNPs) with a minor allele frequency ≥0.1% and imputation quality score ≥0.7 were included (range 7.5-10.5 million per cohort). Analyses were adjusted for age, sex, height, and population substructure. Meta-analysis was performed using a fixed-effects model. Although no genome-wide significant loci were identified, 67 and 60 suggestive loci (p< 5*10-5) were detected for SPPB score and chair-rises time, respectively. Pathway-based analyses indicated significant enrichment of genes affecting negative regulation of calcium channel activity (Bonferroni corrected p-value < 0.05). Sex-stratified gene-based analyses identified clathrin vesicle-associated sec14 protein 1 (CLVS1), significantly associated with chair-rise time in women (p= -1.5*10-7). CLVS1 is highly expressed in the cerebellum, which is involved in postural and motor function control. A larger sample size is needed to confirm and extend our findings, but our results potentially implicate a novel pathway and locus for physical performance in older women.

